# Understanding the Microstructure of Mortars for Cultural Heritage Using X-ray CT and MIP

**DOI:** 10.3390/ma14205939

**Published:** 2021-10-10

**Authors:** Valentina Brunello, Carmen Canevali, Cristina Corti, Tim De Kock, Laura Rampazzi, Sandro Recchia, Antonio Sansonetti, Cristina Tedeschi, Veerle Cnudde

**Affiliations:** 1 Dipartimento di Scienza e Alta Tecnologia (DiSAT), Università degli Studi dell’Insubria, via Valleggio 9, 22100 Como, Italy; sandro.recchia@uninsubria.it; 2 Department of Materials Science, University of Milano-Bicocca, via Roberto Cozzi 55, 20125 Milan, Italy; carmen.canevali@unimib.it; 3 Dipartimento di Scienze Umane e dell’Innovazione per il Territorio (DiSUIT), Università degli Studi dell’Insubria, via Sant’Abbondio, 12, 22100 Como, Italy; cristina.corti@uninsubria.it (C.C.); laura.rampazzi@uninsubria.it (L.R.); 4 Centro Speciale di Scienze e Simbolica dei Beni Culturali, Università degli Studi dell’Insubria, via Valleggio 9, 22100 Como, Italy; antonio.sansonetti@cnr.it; 5 ARCHES (Antwerp Cultural Heritage Sciences), Faculty of Design Sciences, University of Antwerp, Mutsaardstraat 31, 2000 Antwerp, Belgium; tim.dekock@uantwerpen.be; 6 Department of Geology, Pore-Scale Processes in Geomaterials Research (PProGRess)—UGCT, Ghent University, Krijgslaan 281/S8, 9000 Ghent, Belgium; Veerle.Cnudde@UGent.be; 7 Istituto di Scienze del Patrimonio Culturale (ISPC-CNR), via Roberto Cozzi 53, 20125 Milan, Italy; 8 Department of Civil and Environmental Engineering (DICA), Politecnico of Milan, Piazza Leonardo da Vinci 32, 20133 Milan, Italy; cristina.tedeschi@polimi.it; 9 Environmental Hydrogeology, Department of Earth Sciences, Utrecht University, Princetonlaan 8a, 3584 CB Utrecht, The Netherlands

**Keywords:** cement, stone conservation, 3D visualization, µCT, MIP, mortar

## Abstract

In this study, the microstructure of mock-up mortar specimens for a historic environment, composed of different mixtures, was studied using mercury intrusion porosity (MIP) and microcomputed tomography (µCT), highlighting the advantages and drawbacks of both techniques. Porosity, sphericity, and pores size distribution were studied, evaluating changes according to mortar composition (aerial and hydraulic binders, quartz sand, and crushed limestone aggregate). The µCT results were rendered using 3D visualization software, which provides complementary information for the interpretation of the data obtained using 3D data-analysis software. Moreover, µCT contributes to the interpretation of MIP results of mortars. On the other hand, MIP showed significant ink-bottle effects in lime and cement mortars samples that should be taken into account when interpreting the results. Moreover, the MIP results highlighted how gypsum mortar samples display a porosity distribution that is best studied using this technique. This multi-analytical approach provides important insights into the interpretation of the porosimetric data obtained. This is crucial in the characterization of mortars and provides key information for the study of building materials and cultural heritage conservation.

## 1. Introduction

Micro-scale analysis of building materials could provide important information on their structural, physical, and mechanical properties. Microstructure and porosity can be directly observed using several image analysis techniques like scanning electron microscopy or thin section analysis [1,2,3,4,5,6,7,8]; moreover, they can be studied via indirect techniques, such as mercury intrusion porosimetry (MIP) [5,7,8,9,10,11,12,13,14,15,16,17,18,19,20,21], gas sorption, or water absorption [5,8,20,22]. Recently, innovative techniques have become more available, such as microcomputed X-ray tomography (µCT) [6,11,12,13,23,24,25,26,27,28,29,30,31], NMR porosity methods, such as relaxometry mode [8,32,33,34,35] and small angle neutron scattering [8,36], which allow non-destructive sample analysis, including 3D visualization of a material’s inner structure. Even if the non-destructive nature of these techniques is a crucial asset in material characterization, especially in the field of cultural heritage, sampling may be needed in order to improve size and shape assessment.

The aim of this research is the analysis of the porosity properties of mock up samples prepared for the purpose of mimicking historical mortars. Pore size distribution plays a key role, since it is related to moisture transport phenomena, mechanical properties, penetration of polymeric conservation treatments, and compatibility with integration mortars [37,38,39,40,41].

As noted in the literature, MIP is the most used technique to investigate porosity. The Washburn equation is the base principle of MIP analysis. This equation relates the contact angle (θ) formed by a non-wetting liquid (Hg) and a surface of the material under study, with the surface tension (γ) of the liquid itself. All these parameters are connected to the pressure (P) applied in order to obtain the measurement of the equivalent pore radii (r) [23,42,43]: r = −2γcosθ/P [5,7,8,9,10,11,12,13,14,15,16,17,18,19,20,21]. This technique shows some limitations, which have been highlighted in different studies [12,15,16,21,40,42,44,45,46,47,48,49]. One of the most acknowledged biases is the so-called “ink-bottle effect”; when large internal pores are only accessible by narrower pore throats, the intruded volume that determines the pore size distribution misrepresents the size of these large internal pores by the size of their (smaller) throats. MIP analyses could create another problem with respect to the high pressures used, which in some cases, could alter the samples through the formation of cracks and ruptures, which are not easily identified [12]. Moreover, after the analysis, the samples are fully filled with mercury, hindering the possibility of carrying out further analyses on the same sample.

In this research MIP was supplemented with X-ray microcomputed tomography (µCT) to interpret its results, evaluating the advantages and drawbacks of each specific technique, as well as their synergistic effects. µCT allows performing an analysis of materials in a non-destructive way that shows numerous advantages: for example, different (complementary) analyses or dynamic experiments can be carried out on the same sample. Some examples of the application of X-ray µCT analysis on building materials and on the analysis of cement porosity are reported in the literature [8,12,23,50,51,52,53]; Plessis et al. [26] compared different resolutions of µCT analysis using cement samples; Arandigoyen et al. [9,20] studied the application of this technique in depth for the study of porosity and the mechanical properties of cement and lime-based mortars. On the other hand, there are a few papers concerning the study of mortar porosity in the context of heritage studies [12,40,41,54,55,56,57], which are the main focus of this paper. A limitation of this technique regards the spatial resolution, which is physically limited by instrumental features, such as the X-ray source’s spot size, the geometrical magnification, and the detector pixel size [58]. In practice, laboratory systems are often bound to a minimum voxel size just below 1 µm, but most research on stone or cementitious materials is performed with a voxel size of 10^0^–10^2^ µm. 

Munch and Holzer have investigated the fundamental principles that differentiate the results of MIP and imaging techniques, arguing that MIP results represent a continuous spectrum whilst any image analysis (here based on 3D FIB-SEM) is intrinsically based on the discretization of objects with a specific size. Experimental comparisons of MIP and µCT analyses on the same set of samples have been reported in several works on rock porosity, but they are quite neglected in the context of mortar analysis [11,12,21,23,25,42,46]. Hence, they have not yet been applied to a large set of mortar specimens in the context of building and conservation science. This paper is a part of wider research with the aim to compare different analytical techniques for the study of mortars in the heritage field [59,60].

## 2. Materials and Methods

### 2.1. Mock-Up Preparation

Mock-up mortars (Table 1) were made of lime, gypsum, natural hydraulic lime, and cement with quartz sand (standard quartz sand with controlled granulometry and constant mineral composition according to EN 196) and fine and coarse crushed limestone aggregate (grain size fractions 0.063–0.5 mm and 0.7–1.2 mm, respectively). Lime putty was designated as CL 90-Q according to EN 459-1 [61]. The mock-up mortars were prepared according to EN 1015-11 and EN 196 [62,63], as described elsewhere [59,60]. The samples were prepared in a steady environment room, at 20 °C and 65% RH. The specimens with aerial binders were cured under the same environmental conditions for 6 months. The cements and natural hydraulic mortars were cured in a climatic chamber with controlled T and RH at 20 °C at 90%, respectively, according to EN196 [63]. They were considered as the most representative mixtures used as aerial and hydraulic mortars, from past centuries until now. Two cement mortars were studied in order to evaluate modern materials (Table 1). The binder/aggregate ratio was 1:3 (*w*/*w*). The mock-ups were analyzed after 6 months of curing and then studied by means of the following techniques.

### 2.2. Mercury Intrusion Porosimetry (MIP)

Mercury intrusion porosimetry was carried out using Thermo Scientific PASCAL 140 (Thermo Fisher Scientific, Waltham, MA, USA) and PASCAL 240 instruments (Thermo Fisher Scientific, Waltham, MA, USA); the total pressure ranged from 0.1 to 200 MPa, with a resolution of 0.01 MPa below 100 MPa and a resolution of 0.1 MPa from 100 to 200 MPa. The resolution of the volume was 0.1 mm^3^, and the accuracy was >0.2%. The measuring range radius was between 0.0037–0.0075 µm and 35–50 µm. For each specimen, two fragments that were sized around 9 × 10 × 3 mm^3^ were analyzed. For each specimen, two samples were analyzed using this technique.

### 2.3. X-ray Micro-Computed Tomography (µCT)

µCT images were acquired on a custom-built HECTOR scanner at the Centre for X-ray Tomography of Ghent University (UGCT) [64]. This scanner rotates the sample 360° during analysis, and the geometric settings resulted in a voxel size of 10 µm. Images were obtained with an X-ray tube operated at 160 kV and 10 W, equipped with a 1 mm - Al filter to reduce beam hardening and by taking 1401 frames over the 360° [58]. Octopus Reconstruction and Octopus Analysis were used for reconstruction and image analysis, respectively [64,65]. VGStudio^®^MAX software was used for 3D visualization. Image segmentation was performed with a user-dependent dual threshold selection. For the calculation of the sphericity, only voids with at least 3 voxels of equivalent diameter were considered in order to exclude the smallest volumes that could produce errors in the calculation. Octopus Analysis calculated the equivalent diameter as “the diameter of a sphere with the same number of voxels as the object” and the maximum opening as “the diameter of the largest inscribed sphere in the object (only possible if the distance transform is determined)” according to the software manual [66]. In the interpretation of the results, we considered that the volume analyzed for each sample was divided into two parts to reduce computational time of µCT image analysis and the results were averaged. The sample dimensions were approximately 1 cm^3^. The analyzed volume was cylindrical with a diameter of about 9.7 mm and a height of about 8.4 mm (total volume 787.9 mm³).

### 2.4. Environmental Scanning Electron Microscope (ESEM)

Analysis using environmental scanning electron microscopy (FEI/Philips, Hillsboro, OR, USA) and elemental analysis on the samples were performed on polished cross sections using an electronic microscope from FEI/Philips, model XL30 ESEM, in the low vacuum mode (1 torr H_2_O) at a 20 kV acceleration potential, using a backscattered electron detector (BSE). Elemental analysis was performed using an energy dispersive X-ray spectrophotometer from Ametek Element.

## 3. Results

### 3.1. Mercury Intrusion Porosity (MIP)

**Figure 1:**
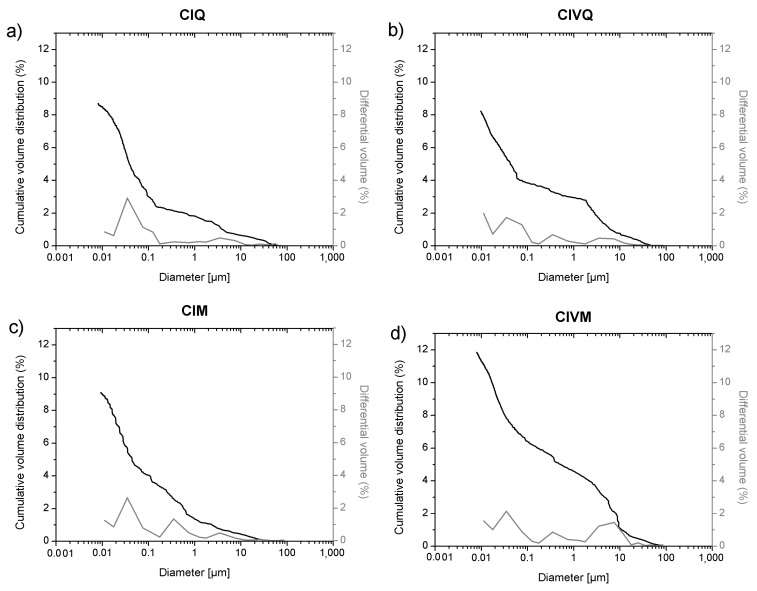
MIP cumulative volume distribution (black) and differential volume (gray) of samples (**a**) CIQ, (**b**) CIVQ, (**c**) CIM and (**d**) CIVM.

**Figure 2:**
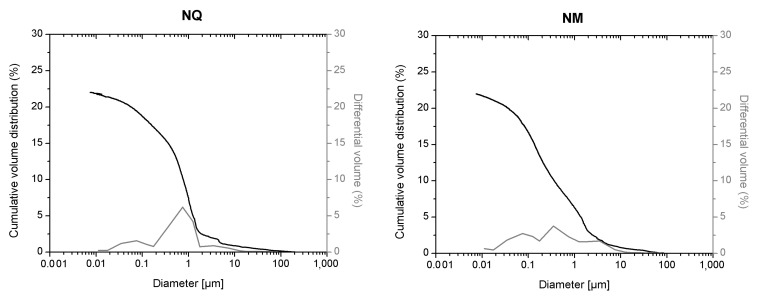
MIP cumulative volume distribution (black) and differential volume (gray) of samples with natural hydraulic lime (NQ, **left**, and NM, **right**).

**Figure 3:**
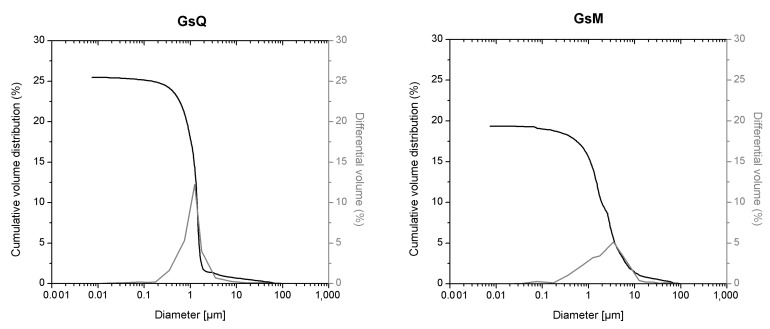
MIP cumulative volume distribution (black) and differential volume (gray) of samples with gypsum (GsQ, **left** and GsM, **right**).

**Figure 4:**
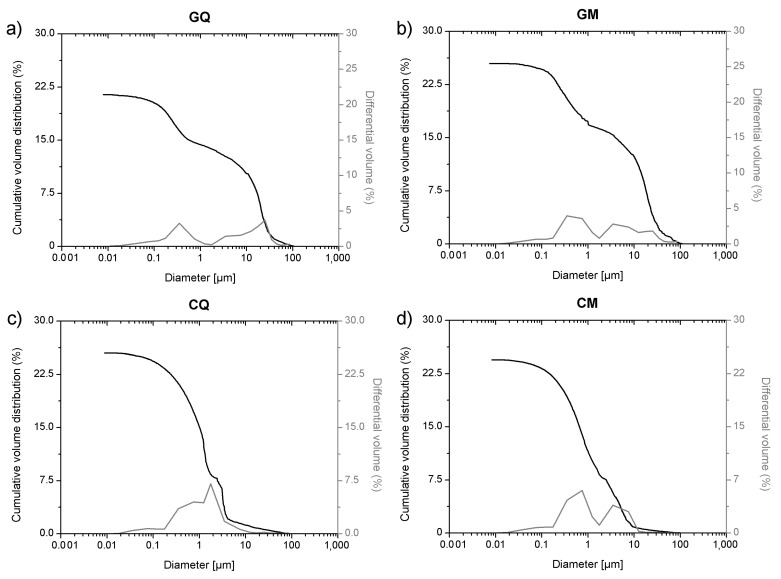
MIP cumulative volume distribution (black) and differential volume (gray) of samples with aerial lime: (**a**) GQ, (**b**) GM, (**c**) CQ and (**d**) CM.

In this study the cumulative porosity, the volume, and the pore diameter size distribution were measured to estimate the total porosity and the pore structure of cured mortar samples (Figure 1, Figure 2, Figure 3 and Figure 4). The results obtained are shown in Table 2. The mortars made by Portland and pozzolanic cements (samples CIQ, CIM, CIVQ, and CIVM) have average pores radii ranging from 0.02 to 0.09 µm, and a total % porosity ranging between 8.7% and 12%. These values are very small if compared to the other mortars (aerial and natural hydraulic mortars). Samples CQ, CM, GsQ, GsM, GQ, GM, NQ, and NM have, in fact, pores radii values ranging from 0.13 to 4.35 µm and the total % porosity varies from 19% to 25.5%.

In Figure 1, the cumulative volume (%) distribution values in relation to the pore size distribution and the relative specific volume of mortars made with cement binder are shown. The four cement mixes show a trimodal pore distribution and the tendency to show low volumes over 10 µm diameters. There are three pore throat diameter zones, distributed as follows:

- between 0.01 µm and 0.1 µm;
- between 0.1 µm and 1 µm;
- between 1 µm and 10 µm.

This trend has also been observed in the samples made with natural hydraulic lime (Figure 2). 

The observed behavior could be related to multiple factors, such as the composition of the binder (hydraulic compounds), or the modalities used to stir the mixes [59,60]. In fact, a porosity ranging from 0.1 to 100 µm is usually connected to capillary pores and contributes to the capillary water transfer, while a porosity lower than 0.1 µm is connected to the presence of sorption pores; in this type of pore water is retained on the surface with no moisture transport. Sorption pores are due to the presence of hydrated hydraulic phases such as CSH (calcium silicate hydrate) in mortars [5,37].

Sorption pores are also found in samples NM and NQ, made with commercial natural hydraulic lime, thus justifying the trimodal pore size distribution (Figure 2). In fact, as other hydraulic mortars, NM and NQ should contain CSH phases, even if their identification by powder X-ray diffraction, infrared, and Raman spectroscopy was difficult [59,60]. With regards to the same range (porosity lower than 0.1 µm), we can see that the curve is flattened in the specimen set composed of aerial mortars (Figure 3 and Figure 4), because of the lack of sorption pores in these materials. Moreover, NM and NQ showed different values of amplitude on the y scale from CIM, CIQ, CIVM, and CIVQ because mortars with natural hydraulic lime display higher porosity than mortars with cement binders.

On the other hand, NM and NQ mixes show lower average pore radii than samples made of aerial binders, but higher than those of the cement specimen. NQ and NM also showed a higher porosity than the cements mortars, with values that were more similar to mortars made with aerial lime as binder (Table 2).

All the aerial and natural hydraulic lime binder specimens showed higher average pores radii than specimens made with cement. Moreover, the total porosity was greater than 19% and the pore radii were higher than those of the cementitious materials. The samples made with gypsum binder (GsQ and GsM) displayed a very narrow pore size distribution, around 1 µm, which is particular and characteristic of this blend (Figure 3) [67,68].

**Figure 5:**
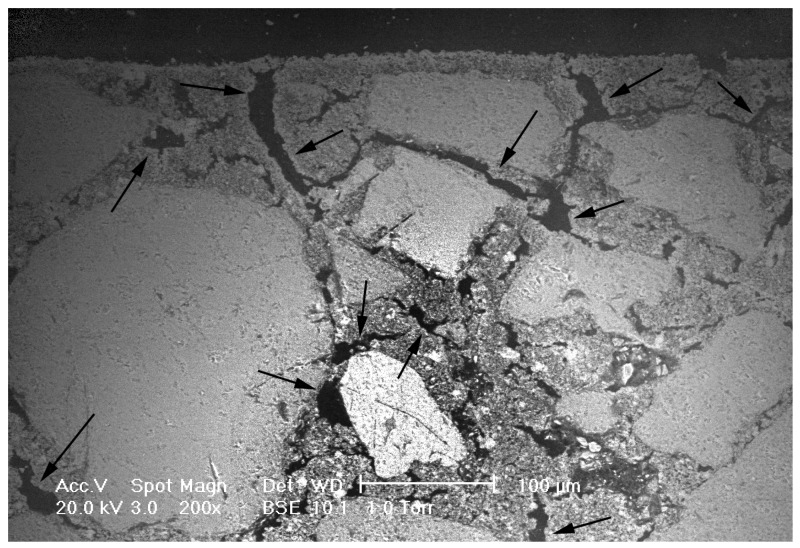
SEM image in back-scattered electron mode of the sample GQ, composed of a lime putty binder with quartz sand aggregate. Cracks are well visible in the matrix (arrows).

Samples GQ, GM, CQ, and CM, composed of lime binders, showed a bi-modal pore size distribution (Figure 4), except for GM that appeared more similar to a trimodal distribution. The total porosity was over 20%. Samples made with hydrated lime (CQ and CM) had lower values of average pore radii (0.44–0.58 µm) than mortars (GQ and GM) made with lime putty (4.34–2.42 µm), although the composition of the two binders was similar. Lime putty samples formed some cracks during the drying process, as clearly shown by ESEM images (Figure 5), which could probably have influenced the results and may be due to rapid hardening or to shrinkage during the drying phase. This phenomenon probably occurs in lime putty mortars because lime putty contains a large amount of free water.

### 3.2. X-ray Computed Tomography Porosity

**Figure 6:**
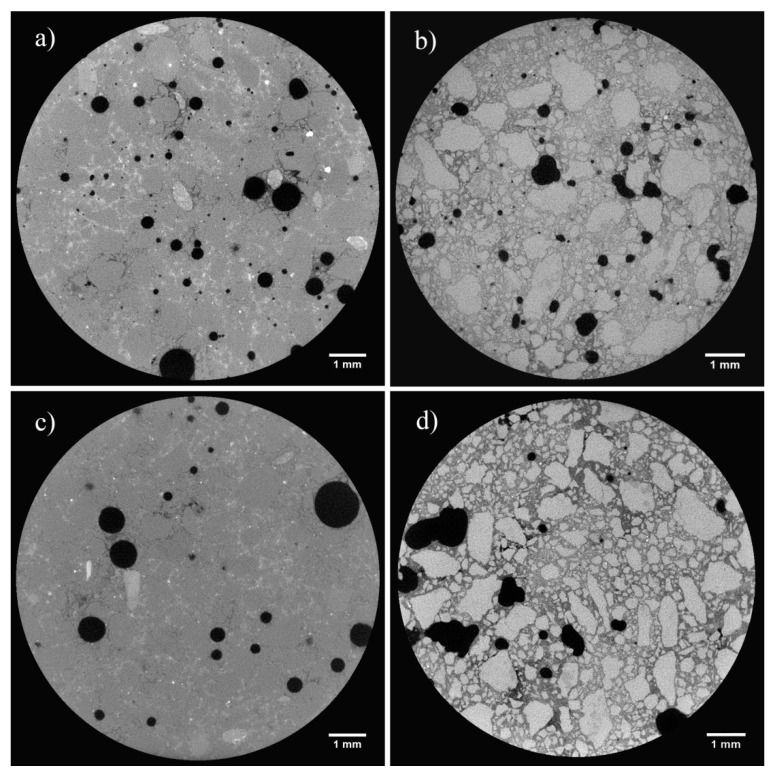
Slices of X-ray tomography of samples (**a**) CIQ, (**b**) CIM, (**c**) CIVQ and (**d**) CIVM.

**Figure 7:**
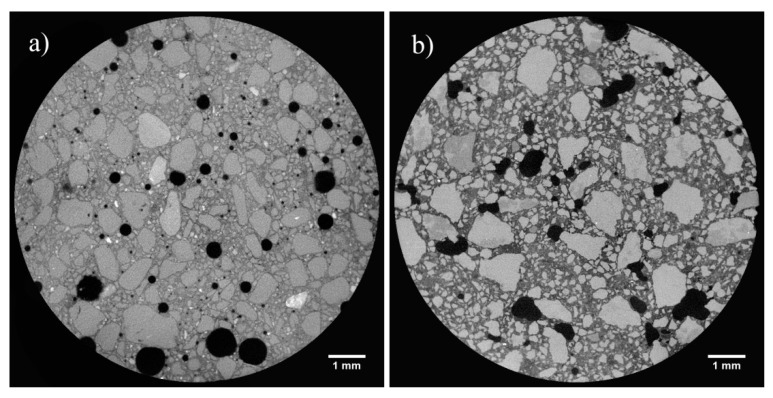
Slices of X-ray tomography of samples (**a**) NQ and (**b**) NM.

The porosity, the maximum opening, the equivalent diameter, and the sphericity are shown in Table 3 and in Figure 6 and Figure 7.

The highest sphericity is observed in samples CIQ, CIVQ, and NQ (0.67, 0.59, and 0.63, respectively). When the sphericity value gets closer to 1, the corresponding object is more similar to a sphere [66]. 

**Figure 8:**
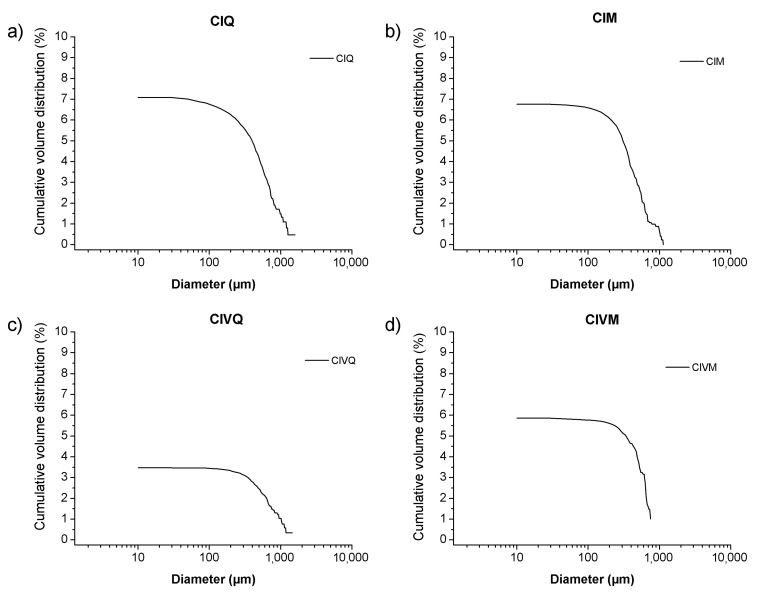
Cumulative porosity distribution of samples: (**a**) CIQ (**b**) CIM (**c**) CIVQ and (**d**) CIVM analyzed with computed X-ray tomography.

Samples prepared with the same binders, but mixed with crushed limestone aggregate, had sphericity values of 0.57 (CIM), 0.46 (CIVM), and 0.47 (NM). These values were lower than the corresponding samples (CIQ, CIVQ, and NQ) prepared with quartz sand. Although porosity is influenced by several factors, in this case, the variability for CIQ, CIVQ, NQ and CIM, CIVM, and NM model samples was probably due to the different aggregates present in the mixtures. The samples made of quartz sand showed, in fact, more spherical pores compared to those containing crushed limestone aggregate (Figure 6 and Figure 7). Probably, the composition and the shape of the aggregate grains influenced the air void distributions inside the specimens. This trend could also be observed in the other samples; in fact, the ones with quartz sand had a higher sphericity if compared to those made with the same binder, but with crushed limestone aggregate (Table 3). Figure 8 shows the cumulative porosity distribution of CIQ, CIM, CIVQ, and CIVM. Based on the analyses, CIQ (7.1%) and CIM (7.4%) had a similar porosity, while CIVQ (3.5%) and CIVM (5.9%) showed different values. This could be the result of heterogeneity, in this size scale of samples, which included, e.g., the air void distribution combined with limits in image resolution. 

**Figure 9:**
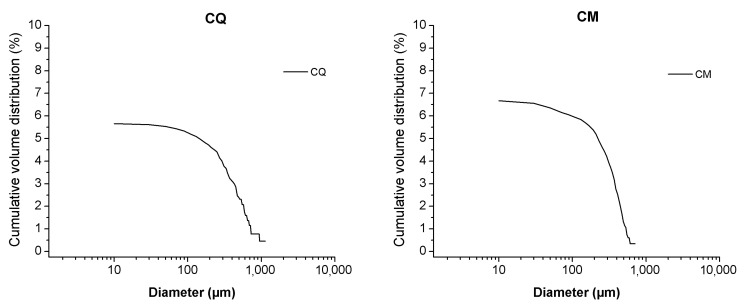
Cumulative porosity distribution of CQ (**left**) and CM (**right**) samples analyzed with computed X-ray tomography.

The cumulative porosity distributions of samples CQ and CM, both containing lime binder, are shown in Figure 9. The total porosity percentage was similar (5.6% and 6%, respectively) and confirmed the similar trend observed for mortars with the same binder. 

The highest porosity recorded by microcomputed tomographic analysis were for specimens GQ, GM, and GsM (18%, 12.9%, and 12%, respectively). Specimens GQ and GM were made using lime putty that, during the setting and hardening processes, created cracks in the samples (Figure 5). The higher porosity values of these samples, compared to the rest of the specimens, was related to the intrinsic nature of the binder. The results are in accordance with those found by Schäfer et al., Hayen et al., Papayianni et al., and Thomson et al. [37,69,70,71], who determined that slaked lime mortars (lime and lime putty) usually have higher total porosities than hydraulic lime mortars.

**Figure 10:**
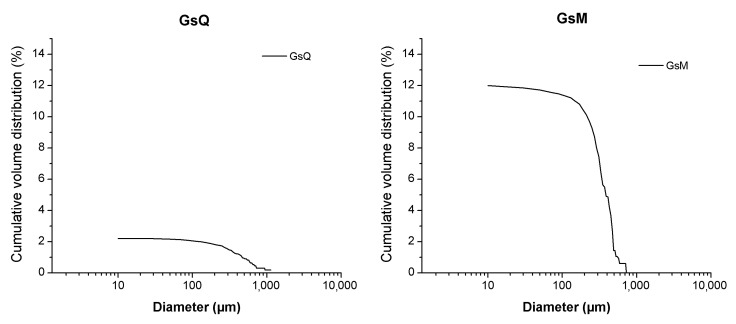
Cumulative porosity distribution of GsQ (**left**) and GsM (**right**) samples analyzed with computed X-ray tomography.

In Figure 10 and Table 3 the differences in the porosity of samples GsQ and GsM (gypsum with quartz and crushed limestone aggregate, respectively) are highlighted. Although they were prepared with the same binder (gypsum), there was an evident difference in the cumulative porosity distribution, which could be explained by the different aggregates present in the mixtures or in some differences in the mortar setting, even if the same environmental conditions and the same mixing procedure were adopted.

**Figure 11:**
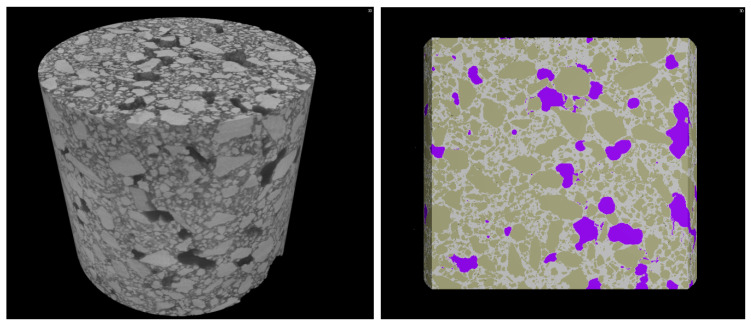
Example of the 3D visualization of sample NM. On the **left**, the gray scale reconstruction cylinder is visible. On the **right**, a section of the cylinder in false color is shown. The pores (violet), aggregate (light brown) and the binder (white) are highlighted. (For interpretation of the references to color in this figure the reader is referred to the web version of this article).

One of the specificities of µCT technique is the capability to visualize the analyzed volume in 3D images, as shown in Figure 11 for sample NM. In fact, µCT allows visualization of pores inside the specimen with a non-destructive approach. This can advance our understanding of the pore structure and absolute values obtained by the different techniques.

### 3.3. Comparison of MIP and µCT Porosity Results

Some general statements should be considered before comparing the results of MIP and µCT analyses. It is important to take into account that the investigated techniques measure pore sizes in two slightly different ranges. The µCT technique allows evaluating the porosity of air voids with a diameter over 10 µm, while the MIP technique evaluates pores (throats) with a diameter less than 90 µm. Thus, the measuring ranges overlap between 10 µm and 90 µm. 

Ink bottle effects in MIP could result in larger pores being counted in the smaller pore sizes. Thus, it can be expected that the total porosity of MIP is correct if we assume that the pore network of the mortars is connected by throats of a 3.7-nm minimum, which is the lowest limit investigated by the MIP technique. With the used configuration, µCT cannot determine pore sizes in the micron and submicron ranges, but the pore and air void sizes that were measured were validated by visual confirmation of the data and images in the 3D models. 

**Figure 12:**
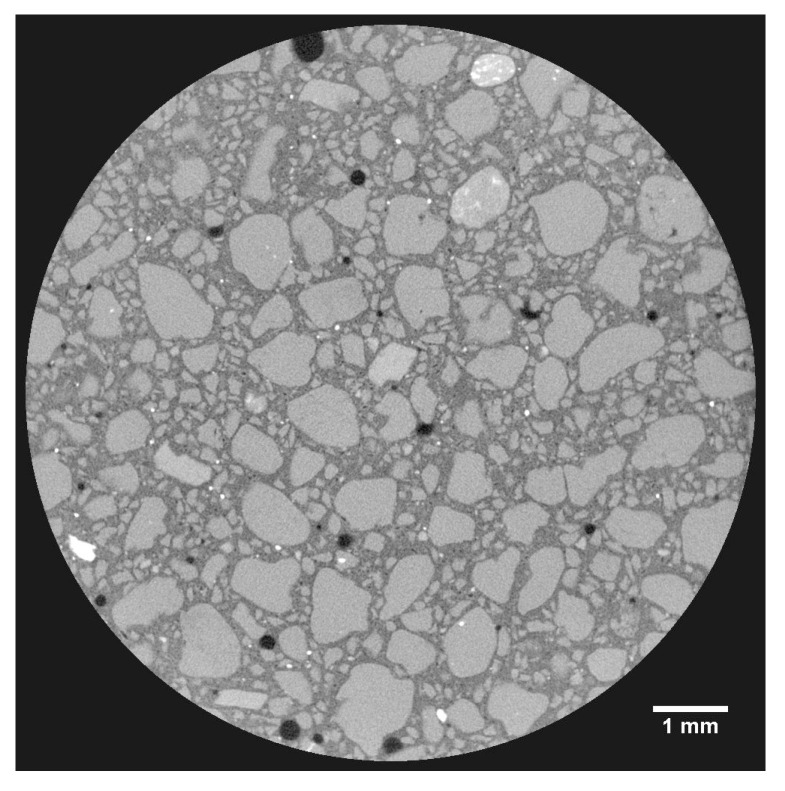
Slice of X-ray tomography for sample NQ.

The first discrepancy was observed in specimens containing gypsum binders, such as sample GsQ, as a remarkable difference between the porosity results obtained by MIP (25.5%) and µCT (2%) was recorded. In this specimen (Figure 3), the critical pore diameter is centered at around 1 µm, hence below the voxel size of the µCT images. These show a centered value of the maximum opening, measured around 28 μm, hence missing larger air voids that are seen in other samples (Figure 12), indicating a unimodal pore size. It can be assumed that the total porosity and the pore size distribution shown by MIP are very accurate, due to the lack of large pores in specimens, which implies a much less significant ink bottle effect that could affect the MIP results. The critical pore diameter is the pore size corresponding to the inflection point in the cumulative mercury volume curve where it begins to increase [72], as shown in Figure 2, Figure 3 and Figure 4.

**Figure 13:**
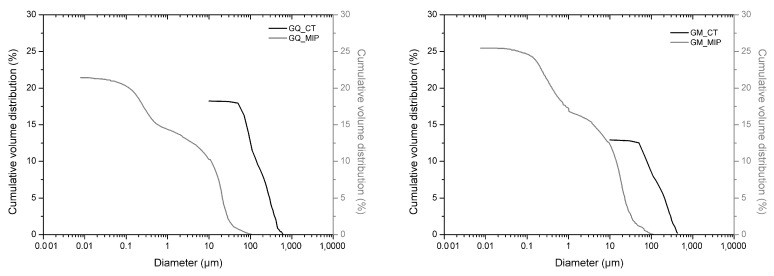
µCT (gray) and MIP (black) cumulative porosity combined graphs of samples GQ (**left**) and GM (**right**).

Some specimens, such as GQ and GM, show µCT porosity values that were 18% for GQ and 14.2% for GM, and MIP values that were 21.8% and 25%, respectively (Figure 13 and Table 4). Pore sizes measured by MIP were below 0.1 µm, probably because the samples have a wider pore size distribution. As can be observed in Figure 13, MIP analysis showed a well-represented pore distribution over 10 µm, where µCT could record the values as well. MIP suggests a bimodal pore size distribution, with modals both in the µCT size range and below the µCT voxel resolution. The MIP data show a cumulative porosity of 10% and 11.8% for GQ and GM, respectively, for pore sizes greater than 10 µm. However, µCT analysis indicated porosities of 18% and 14.2%, respectively. As these pores were validated visually, MIP was assumed to miss approximately 8% and 2.4% in this size range, respectively. This behavior can be explained by two phenomena: (i) the large pores at the surface were not determined; (ii) the ink-bottle effects were present. The former would mean that MIP slightly underestimated the measured total porosity, while the latter would suggest that the cumulative volume of pores <10 µm was overestimated by the absolute difference between both methods, subtracting the errors related to surface pores. 

Air voids might affect the results of cement-based samples (CIQ and CIM, Figure 1 and Figure 8). They display very similar total porosity values measured using MIP and µCT. With regards to MIP analysis, these samples showed that most of their pore sizes were under 1 µm, under the detection limit of µCT; µCT measured air voids that were visually confirmed in the images. Such large air voids are probably connected by submicron throats, which are measured by MIP. Hence, a strong deviation in the measurements occurred, which could be explained by two effects: the total porosity measured by MIP mainly represents the porosity related to the air voids, and the pore size distribution provides a partial deceptive image of the actual pore size volumes. As an alternative, the air voids are inaccessible to mercury because of the small size of the pore connections, or of the ink bottle effect, or because of the presence of closed pores, as observed in previous work [12].

## 4. Discussion

In what concerns MIP analysis of lime mortars (CM, CQ, GM, GQ), the results are comparable to those found by Mosquera et al. [7], in terms of medium radius values and the bimodal distribution. On the contrary, Arandigoyen et al. [20] found a unimodal trend in lime mortars and measured higher porosity values than those discussed in this paper. Moreover, the porosity values found in this research are in accordance with the results determined by Schäfer et al., Hayen et al., Papayianni et al., and Thomson et al. [37,56,69,70,71], who found that lime mortars usually have higher total porosities compared to hydraulic lime mortars.

Gypsum-based mortars (GsQ and GsM) show a narrow distribution around the modal pore sizes of 1 µm and 1.5 µm. These values are in agreement with those shown by Jroundi et al. [68], Freire et al. [73], and Brunello et al. [67], in the case of samples without aggregates. Romera et al. [57] also recorded a very narrow critical pore diameter, but at lower dimensions, if compared to the ones presented here. Moreover, this narrow distribution of gypsum can explain the difference in the results obtained by µCT porosity for the same samples, because the two techniques measured different pore size distributions. This means that µCT allows identifying larger air voids that are connected to the surface only by smaller pore sizes, resulting in ink bottle effects in the MIP data. On the other hand, MIP results give information on this submicron and micron-sized connections, which are currently lacking in µCT data. 

Concerning the cement mortar samples, the total porosity calculated by MIP and µCT is similar to those found by Cnudde et al. [12] and Plessis et al. [26] in different cement pastes. The main difference is in the trimodal distribution found in this research, contrary to the research by Cnudde et al. [12]. On the other hand, Arandigoyen et al. [9] [20] recorded a MIP porosity value around 20% for cement mortars specimens, higher than the data obtained here, while the porosity results for lime mortars are in accordance with the values of this research. Cook et al. [19] found a unimodal pore size distribution as well, which tends to be less evident with an increase in curing time. Moreover, most of the identified pores are linked to the presence of sorption pores, characteristic of cement pastes, as also the case in the cement specimens in Figure 1. These differences in pore size distribution could be due to different reasons, such as the raw material, the water/binder ratio, the curing environment, the aggregate nature, etc. [19,37]. 

The comparison of porosity values of natural hydraulic 3.5 mortar (NQ and NM) to the values recorded by Gulotta et al. [39] shows that the total porosity is over 20% in both cases. In Gulotta et al. [39], the value was 27.08% while in NQ and NM the values are 21.9% and 22%, respectively. On the other hand, there are greater differences in the average pore radii among mortars studied by Gulotta (0.43 µm for M3 mortar) and those investigated here, although they are in the same order of magnitude (0.34 µm for NQ and 0.13 µm for NM). However, the main difference is that Gulotta et al. observed a unimodal pore size distribution starting under 1 µm, while, in this paper, a trimodal distribution is reported.

In this paper MIP analysis of hydraulic mortars (CIQ, CIM, CIVQ, CIVM, NQ, and NM) shows a trimodal pore size distribution. Pores under 0.1 µm are particularly important because they are connected to sorption pores, indicating the presence of hydrated hydraulic phases like CSH [5,37]. MIP results suggest the presence of these phases also in natural hydraulic lime specimens, which can be hardly characterized by traditional analytical techniques, such as infrared, Raman spectroscopy and X-ray powder diffraction. Moreover, MIP data of gypsum-based mortars give very accurate information, correctly characterizing the pore size distribution. On the other hand, the presence of air voids and small capillary connections for the other mortars should be taken into account.

## 5. Conclusions

To the best of our knowledge, this is the first study that compares two porosimetric techniques on a set of mortar model samples to evaluate the potentials of micro-scale analyses for characterizing historical mortars prepared using different technologies and raw materials. 

The ability of the two techniques in the characterization of total porosity was highlighted and studied with interesting results. For example, the characteristic pore size distribution of gypsum mortars, the porosimetric properties of natural hydraulic lime in comparison with cement mortars, and the pore size distribution of lime mortars, together with the characteristic sphericity of cement pastes, were successfully determined.

µCT correctly identifies and characterizes bigger pores and provides their spatial distributions. Some of them are also possibly measured by MIP, but they were connected with the outer environment with a small throat and thus classified with lower pore size diameters.

The limits of the two techniques should be considered when interpreting the overall results, especially analyzing materials with a wide difference in pore size distribution like building materials. The µCT is non-destructive, thus it is possible to carry out several measures on the same sample after the scan, and it is able to give the three-dimensional reconstruction of the analyzed volume, showing also the internal structure of the sample. In the case of mortars, it could provide visualization of pores, air voids, aggregate, and binder distributions inside the sample.

Thus, we can affirm that these two techniques give complementary information, with the wide range of pores that could be detected. Both for the study of the mortars production techniques and for the application of conservation treatments, such as desalination, consolidation or surface cleaning treatments, a multi-analytical approach should include µCT as this technique provides important complementary information related to 3D structure and 3D pore analysis. This research could be of great interest to conservation scientist involved in the challenging description of structural and mechanical properties of mortars.

In what concerns future developments, the measure of the porosity of the same set of specimens could be compared with other techniques, such as gas or water absorption, NMR methods, microscopical methods, such as optical, electron microscopes or dimensional and surface roughness measurement using new technologies.

## Figures and Tables

**Table 1:** List of the mortar mock ups.

Mortars				
Binder	Aggregate	Water/Binder (*w*/*w*)	B/A (*w*/*w*)	Label
Lime putty	quartz sand	-	1:3	GQ
Hydrated lime	quartz sand	67:100	1:3	CQ
CEM I	quartz sand	50:100	1:3	CIQ
CEM IV	quartz sand	50:100	1:3	CIVQ
Gypsum	quartz sand	55:100	1:3	GsQ
NHL 3.5	quartz sand	55:100	1:3	NQ
Lime putty	crushed limestone aggregate	-	1:3	GM
Hydrated lime	crushed limestone aggregate	67:100	1:3	CM
CEM I	crushed limestone aggregate	50:100	1:3	CIM
CEM IV	crushed limestone aggregate	50:100	1:3	CIVM
Gypsum	crushed limestone aggregate	55:100	1:3	GsM
NHL 3.5	crushed limestone aggregate	55:100	1:3	NM

Legend: CEM I, Portland cement type I; CEM IV, pozzolanic cement type IV; NHL 3.5, natural hydraulic lime.

**Table 2:** Results of the MIP analysis. (Note that “M” is the abbreviation for crushed limestone aggregate while “Q” is the abbreviation for quartz sand).

	MIP Results			
Binder Type	Sample Label	Total Cumulative Volume (mm^3^/g)	Average Pore Radius (μm)	Total Porosity (%)
Lime putty	GQ	126.4	4.35	21.8
	GM	134.0	2.42	24.7
Hydrated lime	CQ	133.3	0.58	22
	CM	133.2	0.44	25
CEM I	CIQ	49.3	0.02	8.8
	CIM	46.1	0.03	9.4
CEM IV	CIVQ	45.5	0.02	8.7
	CIVM	60.7	0.09	12
Gypsum	GsQ	146.5	0.65	25.5
	GsM	109.9	0.92	19
NHL3.5	NQ	121.0	0.34	21.9
	NM	125.5	0.13	22

**Table 3:** Results of the microcomputed tomography analyses (Note that “M” is the abbreviation for crushed limestone aggregate while “Q” is the abbreviation for quartz sand).

	µCT Results				
Binder Type	Sample Label	Porosity Total Volume (%)	Equivalent Diameter (µm)	Maximum Opening (µm)	Sphericity
Lime putty	GQ	18	50.6	28.8	0.52
	GM	12.9	59.8	28	0.47
Hydrated lime	CQ	5.6	43.7	25	0.54
	CM	6	37.1	20	0.49
CEM I	CIQ	7	46	32.2	0.67
	CIM	7.45	93.4	58.5	0.57
CEM IV	CIVQ	3.5	90.5	65.7	0.59
	CIVM	5.9	33.9	19.5	0.46
Gypsum	GsQ	2	45.9	28.1	0.56
	GsM	12	26	14.5	0.48
NHL 3.5	NQ	6	62.4	46.7	0.63
	NM	7	21.7	13.95	0.47

**Table 4:** Porosity results of MIP and X-ray µCT.

	MIP	µCT
Sample Label	Total Porosity (%)	Total Porosity (%)
CIQ	8.8	7
CIM	9.4	7
CIVQ	8.7	3.5
CIVM	12	5.9
CQ	22	5.6
CM	25	6
GQ	21.8	18
GM	24.7	12.9
GsQ	25.5	2
GsM	19	12
NQ	21.9	6
NM	22	7

## Data Availability

The data presented in this study are available on request from the corresponding author.
